# Can enriching emotional intelligence improve medical students’ proactivity and adaptability during OB/GYN clerkships?

**DOI:** 10.5116/ijme.5658.0a6b

**Published:** 2015-12-26

**Authors:** Stephanie H. Guseh, Xiaodong P. Chen, Natasha R. Johnson

**Affiliations:** 1 Department of Obstetrics, Gynecology and Reproductive Biology, Harvard Medical School, Brigham and Women's Hospital, Boston, Massachusetts, USA

**Keywords:** Emotional intelligence, obstetrics and gynecology, resident coach, clerkship

## Abstract

**Objectives:**

The purpose of this pilot study was to examine our hypothesis that enriching workplace emotional intelligence through resident coaches could improve third-year medical students’ adaptability and proactivity on the Obstetrics and Gynecology clerkship.

**Methods:**

An observational pilot study was conducted in a teaching hospital. Fourteen 3rd year medical students from two cohorts of clerkships were randomly divided into two groups, and equally assigned to trained resident coaches and untrained resident coaches. Data was collected through onsite naturalistic observation of students’ adaptability and proactivity in clinical settings using a checklist with a 4-point Likert scale (1=poor to 4=excellent). Wilcoxon rank-sum test was used to compare the differences between these two groups.

**Results:**

A total of 280 data points were collected through onsite observations conducted by investigators. All (n=14) students’ adaptability and proactivity performance significantly improved from an average of 3.04 to 3.45 (p=0.014) over 6-week clerkship. Overall, students with trained resident coaches adapted significantly faster and were more proactive in the obstetrics and gynecology clinical setting than the students with untrained coaches (3.31 vs. 3.24, p=0.019).

**Conclusions:**

Findings from our pilot study supported our hypothesis that enriching workplace emotional intelligence knowledge through resident coaches was able to help medical students adapt into obstetrics and gynecology clinical settings faster and become more proactive in learning. Clerkship programs can incorporate the concept of a resident coach in their curriculum to help bridge medical students into clinical settings and to help them engage in self-directed learning throughout the rotation.

## Introduction

Emotional Intelligence (EI), which usually includes adaptability and proactivity as key components, is the ability to identify, process, control, and manage the emotions of oneself and others.^1^ Many studies have shown that measurements of EI tended to correlate with academic and professional success – including in the medical setting and workplace.^2^ There is increasing interest in defining EI within the medical sphere as it is naturally related to the new Accreditation Council for Graduate Medical Education (ACGME) core competencies of professionalism, patient care, and interpersonal and communication skills.^3^ Some authors have suggested that an assessment of EI should be included as part of the residency application process.^4^ Although many studies have proposed incorporating EI into the medical curriculum, few have described specific methods of enhancing the EI of medical students during their third year clerkships.^5^^-^^6^

Adaptability, which refers to an ability to change oneself to adjust to occurring changes in the environment, is one component of the self-management sphere of workplace emotional intelligence (WEI) that is critical for success during medical school clerkships.^7^ The complex and dynamic nature of the medical environment often makes medical students feel stressed when rotating in a new clerkship. Clerkship students who are in distress may experience cognitive changes that affect their learning. Thus, if medical students are able to rapidly adapt to multiple different care teams as well as various clinical settings, they would be able to integrate themselves into the medical culture, become less stressed, but more active participants, and be well prepared for their learning in OB/GYN.^8^ In addition, a recent study showed that adaptability was one domain of EI in which resident physicians score lower than the general population – thus highlighting the need for specific training in medical school curricula.^9^

Proactivity, which refers to acting in advance of a future situation rather than just reacting, is another component of WEI about self-starting and being change-oriented in order to enhance personal or organizational effectiveness.^10^^,^^11^ Proactive behaviors in the workplace include seeking feedback,^12^^-^^14^ actively adapting to new environments,^15^^-^^17^ taking initiative in pursuing personal and organizational goals,^18^ and implementing ideas and solving problems.^19^ In medical education, proactive medical students usually do not require prompting to complete assigned tasks or require detailed instructions. Students’ proactive behaviors during the clerkship, thus, are believed to have a positive influence on their learning experience.

Although many studies about WEI have been conducted in the medical environment, few of them focus on effective methods to deliver WEI knowledge to medical students during clerkships. At academic teaching hospitals, residents play a critical role in the education of medical students as they rotate through various clerkships. Residents have been identified as having a significant impact on students’ ability to integrate into the medical system and adapt to specific cultural patterns within medicine.^20^^,^^21^ However, as these abilities could be difficult to teach in a group format as well as students’ diverse clerkship schedules, we hypothesized that utilizing resident coaches, in which residents work one-on-one with medical students directly or remotely, we could deliver WEI knowledge to students and help them adapt faster and become more proactive in the OB/GYN clinical setting during the clerkship. Therefore, we conducted this study to pilot test our hypothesis that enriching students’ workplace emotional intelligence (WEI) knowledge through resident coaches could improve medical students’ adaptability and proactivity on the OB/GYN clerkship.

## Methods

### Study design

This was an observational study conducted during the academic year 2013-2014 within the OB/GYN Medical Student Clerkship at Brigham and Women’s Hospital and Integrated Residency Program at Brigham and Women’s Hospital and Massachusetts General Hospital, which both are teaching hospitals affiliated with Harvard Medical School. The OB/GYN clerkships are 6 weeks (3 consecutive weeks focusing on OB and 3 consecutive weeks on GYN) in length for third-year Harvard Medical School students; residency training is 4 years for OB/GYN residents in these institutions. The OB/GYN clerkship consists of 6 to 7 students who rotate through different clinical settings, including: labor and delivery, operating room, inpatient, gynecology oncology, and ambulatory clinics over the course of the 6 weeks.

### Resident coach program

Based on prior medical student needs assessment and resident feedback, the OB/GYN clerkship leadership in conjunction with administrative OB/GYN chief residents initiated the Resident Coach Program in the academic year 2012-2013 as part of the Resident-as-Teacher (RaT) Curriculum for third-year medical students. The main goal of the Resident Coach Program is to provide students with one-on-one support and guidance from residents in person or remotely during their clerkship. Each student is assigned a second or third-year OB/GYN resident as their coach for the 6-week clerkship. The resident coaches follow a standardized job description, which consists of an initial meeting and several check-in points over the course of the 6 weeks, provide students with educational resources, and culminate with aiding in oral examination and shelf examination preparation in the final week. The resident coach is available for any other guidance or support (e.g. suturing, knot tying, patient presentation practice) requested by the student. Given the well-established successful resident coach program in our OB/GYN clerkship, it was chosen as the pilot test platform to deliver the WEI knowledge to medical students.

### Study participants

All of 14 third-year clerkship students (6 female students and 8 male students) and 14 PGY2 and PGY3 resident coaches participated over the course of two 6-week clerkships in this pilot study. To decrease the potential selection bias, all participating students needed to demonstrate similar pre-clerkship academic performance. Individual OB/GYN residents from the 2nd postgraduate year (PGY2) and the 3rd postgraduate year (PGY3) were randomly assigned as resident coaches to each 3rd medical student who participated in the OB/GYN clerkship as part of the already established Resident Coach Program described above. These resident coaches were randomly divided into two groups: resident coaches from group 1 were trained and instructed to teach students WEI, while coaches from the other group (group 2) were not. The WEI training curriculum, which included introductory of clinical environment and resources, the student’s role on the working team, and a list of recommended clinical learning tasks, were provided to residents from group 1. Clerkship expectations for resident coaches (e.g. scheduled times to “check-in” during the clerkship) and instruction regarding adequate support for the students were also included as general training components for resident coaches in both groups.

This study received Institutional Review Board (IRB) approval from both Harvard Medical School and Brigham and Women’s Hospital.

### Data collection and analysis

Data was collected through onsite naturalistic observations of students’ clerkship performance in different clinical settings with an evaluation checklist which was derived from validated EI literature.^1^^,^^22^^-^^24^ According to the taxonomy of adaptive performance,^23^ ten checklist rating items from 5 dimensions were developed: physically and environmental adaptability, interpersonal adaptability, cultural adaptability, dealing with uncertain work situations, and learning work tasks/technologies/procedures (Table 1).

**Table 1:** Workplace Emotional Intelligence (WEI) checklist items

Category	Checklist Items	Definition
Physically and environmental adaptability	Locate patient	Able to find the patient/exam room successfully without asking for help
	Movement	Able to adjust body movements instantly based on the clinical setting without reminder
Interpersonal adaptability	Self-Introduction	Able to greet and/or self-introduce to the team without reminder
	Call Names	Able to remember and call most team members’ names
Cultural adaptability	Team Structure	Able to identify the right team member to ask for appropriate support
	Setting	Able to demonstrate understanding of the current clinical setting (e.g. patient’s need for privacy, schedule)
Dealing with uncertain work situations	Reaction	Able to demonstrate understanding of his/her role, join the team, and provide appropriate support without residents’ and/or faculty’s prompting
Learning work tasks, technologies, and procedures	Understand Task	Able to respond to the order quickly and accurately without prompting
	Discuss Task	Able to identify learned knowledge for the task and discuss alternatives without prompting
	Perform Task	Able to perform and complete the task successfully without asking for help

A 4-point Likert scale (1=poor), (4=excellent) which was derived from an existing adaptability measurement inventory with detailed rating description was applied.^25^ Expert validation was conducted with 2 faculty members, who had sufficient experience in clerkship students’ cognitive behaviors and psychometric evaluation, to ensure the checklist items’ clarity and relevance to the construct. Cognitive pre-testing was then applied to collect feedback from 2 residents and 1 faculty to ensure potential participants interpreted the checklist items as they were intended.

All observations were conducted by a medical educator, non-coach residents, or faculty members. Each student was observed at least twice: once towards the midpoint of the first 3 weeks of the clerkship and once towards the midpoint of the second 3 weeks. All observers were contacted prior to the data collection. If the observer agreed to participate in the study, the checklist would be sent out. All the observers returned the checklists to the investigator within the assigned time frame (e.g. Tuesday to Thursday of the 2nd week). Data from observers was then aggregated into an excel file. Wilcoxon Signed-Rank test was used to compare the differences of students who had WEI trained coaches to those who had untrained coaches depending on the data distribution. P values less than 0.05 were considered statistically significant.

## Results

A total of 14 third-year medical students (6 female students and 8 male students) and 14 PGY2 and PGY3 resident coaches participated over the course of two 6-week clerkships in this pilot study. All participating students demonstrated similar pre-clerkship academic performance. Each student was randomly assigned to either a PGY 2 or PGY 3 OB/GYN resident coach according to the RaT resident coach curriculum. A total of 280 data points were collected through onsite observations.

All of the medical students’ adaptability and proactivity performance significantly improved from an average of 3.04 during the first 3 weeks of the clerkships to 3.45 during the last 3 weeks of the clerkships (P=0.014) over two 6-week clerkships (Table 2).

**Table 2:** Overall Scores of Medical Students’ Adaptability and Proactivity in the 6-week OB/GYN Clerkship

Category	Checklist item	First 3 weeks	Second 3 weeks	Difference
Physically and environmental adaptability	Locate patient	3.14	3.69	0.55
	Movement	3.25	3.63	0.38
Interpersonal adaptability	Self-introduction	3.12	3.63	0.51
	Call names	3.21	3.23	0.02
Cultural adaptability	Team structure	3.81	3.75	-0.06
	Setting	2.92	3.39	0.47
Dealing with uncertain work situations	Reaction	2.98	3.52	0.54
Learning work tasks, technologies, and procedures	Understand task	3.13	3.32	0.19
	Discuss task	2.69	3.18	0.49
	Perform task	2.82	3.15	0.33
Average		3.04	3.45	0.41^*^

Note: During the 6-week OB/GYN clerkship, 3 consecutive weeks usually focus on OB context and 3 consecutive weeks on GYN context. Overall scores calculated over 2 consecutive 6 week clerkships for students in both Group 1 and Group 2. *p value = 0.014 calculated by Wilcoxon Signed-Rank test.

Medical students demonstrated substantial improvement in dealing with uncertain work situations (2.98 vs. 3.52) when comparing their ratings in the first 3 weeks of the clerkships to the second 3 weeks of the clerkships. Moderate-to-substantial improvements were also observed in medical students’ “physically and environmental adaptability” and “learning work tasks, technologies, and procedures” (Table 2). Overall ratings for the adaptability and proactivity in groups 1 (WEI trained) and 2 (non-WEI trained) are shown in Table 3. Across both 6-week clerkships, it is notable that the average performance of students in group 1 was significantly better than the students in group 2 (3.31 vs. 3.24, P=0.019). Students in group 1 tended to show better adaptability and proactivity in four tasks: “locating the patient (3.54 vs. 3.33)”, “movement (3.58 vs. 3.27)”, “understanding the setting (3.35 vs. 3.00)”, and “discussing tasks (3.38 vs. 3.05)”. Students in group 2 were more likely to be proficient at: “knowing team structures” and “performing tasks” during the 6-week clerkship periods. No substantial differences between the two groups in the following tasks were observed: “self-introduction”, “calling team members’ names”, “reacting to uncertainty”, and “understanding tasks”.

**Table 3:** Overall scores of medical students’ adaptability and proactivity in the 6-week OB/GYN clerkship between groups

Category	Checklist item	Group 1	Group 2	Difference
Physically and environmental adaptability	Locate patient	3.54	3.33	0.24
	Movement	3.58	3.27	0.31
Interpersonal adaptability	Self-introduction	3.36	3.36	0.00
	Call names	3.19	3.25	-0.06
Cultural adaptability	Team structure	3.70	3.88	-0.18
	Setting	3.35	3.00	0.35
Dealing with uncertain work situations	Reaction	3.27	3.18	0.09
Learning work tasks, technologies, and procedures	Understand task	2.97	2.89	0.08
	Discuss task	3.38	3.05	0.33
	Perform task	2.74	3.23	-0.49
Average		3.31	3.24	0.07^*^

Note: Overall scores calculated over 2 consecutive 6 week clerkships. Scores based on WEI instrument using 4 point Likert scale; 1=poor to 4=excellent; Group 1- Students paired with Resident Coaches trained in Workplace Emotional Intelligence (WEI), Group 2- Students paired with Resident Coaches not trained in WEI. *p value = 0.019 calculated by Wilcoxon Signed-Rank test.

## Discussion

The aim of our study was to investigate whether enriching WEI knowledge through resident coaches could improve students’ adaptability and proactivity on their OB/GYN clerkship. Students who were paired with trained resident coaches (Group 1) demonstrated significantly higher levels of overall adaptability and proactivity within the 6 week clerkships. Our results suggested that trained resident coaches provided a feasible platform to deliver WEI knowledge to medical students when entering a new clerkship and help students adapt faster and become proactive during the OB/GYN clerkship.

The ability to quickly adapt to a physical environment is a key aspect of effective performance.^23^ OB/GYN uniquely combines multiple clinical settings, including labor and delivery, operating room, outpatient, and inpatient, thus students face bigger challenges of adapting quickly and effectively to different clinical settings than in other core clerkships. In order to maximize their learning potential and to promote proactive self-directed learning, enriching students’ WEI is essential. Our findings revealed that WEI knowledge was not only feasible to be delivered through trained resident coaches, but also able to help medical students improve their physical and environmental adaptability, such as locating patients and appropriately adjusting their movement, and thus immerse themselves into the OB/GYN culture and settings at our hospital. Results from this study also indicated that students with enriched WEI knowledge engaged in seeking novel ways to solve problems in a changing situation which was consistent with the existing literature.^23^^,^^26^ In addition, students with WEI-trained coaches demonstrated superior performance in the categories of cultural adaptability and learning work tasks. Thus, the WEI knowledge imparted through trained resident coaches likely contributed to the students’ adaptability and proactivity during the OB/GYN clerkship.

Although students in group 1 outperformed those in group 2 in a number of categories on the checklist, it is interesting that the students in group 2 received higher ratings in “identifying team structure” and “completing a task”. According to the literature of situated learning, expertise development, and learning curves,^27^^-^^29^ a possible explanation may be that medical students with untrained resident coaches (group 2) became more proficient at independently locating helpful resources through self-directed exploration of the environment. Without adequate WEI knowledge about our OB/GYN environment and learning context, these students would have to rely on themselves to adapt to the environment, and consequently would be expected to demonstrate somewhat delayed learning progress. They might rely more on identifying team structure in order to find appropriate support to accelerate their learning and adaption in different clinical environments. Based on the learning curve model, these students might become more efficient after they ultimately achieve a steep acceleration in learning. As a result, they might perform tasks better than their WEI-trained counterparts during the study period. This finding also leads to an important question for medical education regarding the balance of guided support versus independence for third year medical students during clerkships. Future studies are needed to explore how resident coaches and WEI knowledge might influence medical students’ skills acquisition, learning development, and progressive independence during clerkships.

Our pilot study has demonstrated the possibility and practicability of using resident coaches to help medical students adapt into OB/GYN clinical settings faster and to become more proactive in clerkship learning through enriching their WEI knowledge. However, it has several limitations. As this was a single-institution pilot study and had small participant numbers, we did not expect to demonstrate significant effects and generalizability in our outcome measures. Likewise, there is an increased likelihood of false negative results of statistical data analysis as our study had limited sample size. In addition, our findings may be influenced by limited representativeness as well as social and personal bias. All medical students voluntarily participated in the study, but they may not represent a full population of 3rd year medical students. Although standardized checklists with detailed rating descriptions were used and non-coach residents were applied to data collection, the ratings might be subject to the observers’ biases, influenced by students’ social desirability and personalities. However, our findings were still able to test the hypothesis of our study and provided empirical pilot data for other clerkship programs to reference. Future studies with larger sample sizes and further replication of these findings in other institutions with multiple evaluators would be a beneficial next step.

## Conclusions

Enriching WEI knowledge through resident coaches is a practical and effective method to improve medical students’ adaptability and proactivity in learning during the OB/GYN clerkship, which is paramount to the medical education experience. Clerkship programs can easily embrace resident coaches into their clerkship curriculum to boost students’ preparedness, equip them with WEI knowledge, and guide them to carry out self-directed learning throughout the clerkship rotations.

### Conflict of Interest

The authors declare that they have no conflict of interest. 
